# Qualitative and Quantitative Phytochemical Analysis of Different Extracts from *Thymus algeriensis* Aerial Parts

**DOI:** 10.3390/molecules23020463

**Published:** 2018-02-20

**Authors:** Nassima Boutaoui, Lahcene Zaiter, Fadila Benayache, Samir Benayache, Simone Carradori, Stefania Cesa, Anna Maria Giusti, Cristina Campestre, Luigi Menghini, Denise Innosa, Marcello Locatelli

**Affiliations:** 1 Unité de recherche Valorisation des Ressources Naturelles, Molécules Bioactives et Analyses Physicochimiques et Biologiques, Université Frères Mentouri, Constantine 1, Route d’Aïn El Bey, 25000 Constantine, Algérie; boutaoui.nassima@gmail.com (N.B.); lahcene.zaiter@yahoo.fr (L.Z.); fbenayache@yahoo.fr (F.B.); sbenayache@yahoo.com (S.B.); 2 Department of Pharmacy, University “G. d’Annunzio” of Chieti-Pescara, Via dei Vestini 31, 66100 Chieti, Italy; cristina.campestre@unich.it (C.C.); luigi.menghini@unich.it (L.M.); marcello.locatelli@unich.it (M.L.); 3 Dipartimento di Chimica e Tecnologie del Farmaco, Sapienza Università di Roma, P.le Aldo Moro 5, 00185 Rome, Italy; stefania.cesa@uniroma1.it; 4 Dipartimento di Medicina Sperimentale, Sapienza Università di Roma, P.le Aldo Moro 5, 00185 Rome, Italy; annamaria.giusti@uniroma1.it; 5 Facoltà di Bioscienze e tecnologie agro-alimentari e ambientali, Università di Teramo, Via Renato Balzarini 1, 64100 Teramo, Italy; dinnosa@unite.it

**Keywords:** color analysis, pigments, MAE, HPLC-PDA, SFE, *Thymus algeriensis*

## Abstract

This study was performed to evaluate the metabolite recovery from different extraction methods applied to *Thymus algeriensis* aerial parts. A high-performance liquid chromatographic method using photodiode array detector with gradient elution has been developed and validated for the simultaneous estimation of different phenolic compounds in the extracts and in their corresponding purified fractions. The experimental results show that microwave-assisted aqueous extraction for 15 min at 100 °C gave the most phenolics-enriched extract, reducing extraction time without degradation effects on bioactives. Sixteen compounds were identified in this extract, 11 phenolic compounds and five flavonoids, all known for their biological activities. Color analysis and determination of chlorophylls and carotenoids implemented the knowledge of the chemical profile of this plant.

## 1. Introduction

The genus *Thymus* belongs to the Lamiaceae family, which comprises about 400 genera. They are mainly herbaceous, perennials, small shrubs occurring within the Mediterranean region, which is the center of the entire genus, and are also characteristic in Asia, Southern Europe and North Africa [1]. Historically, the aerial parts of *Thymus* species, rich in volatile constituents, have been commonly used as herbal teas, condiments and spices. In addition, they have shown many ethnomedicinal properties such as tonic, carminative, digestive, antispasmodic, antimicrobial, antioxidant, antiviral, and anti-inflammatory activities [2]. *Thymus* leaves extracts, despite their frequent use as spice and infusions, are used in traditional medicine as astringent, expectorant, antiseptic, anti-rheumatic, diuretic, analgesic and cicatrizing agents. Thyme can also be used as a phytotherapy agent in veterinary (antispasmodic, antiseptic and digestive); it is applied as feed additives and for treating diseases of pets and farm animals [3,4].

*Thymus algeriensis* Boiss. & Reut. (*Thymus hirtus* Willd. ssp. *algeriensis*) is the most widespread North African species. It is endemic in Libya, Tunisia, Algeria and Morocco. *T. algeriensis* is largely used, fresh or dried, mainly as a culinary herb. On the other hand, the species is used in traditional medicine in respiratory disorders, against illnesses of the digestive tube and as anti-abortive [5,6,7,8]. The chemical composition of its essential oil has been previously studied with an exclusive attention to the volatile components, although results of its biological activity are limited [9,10,11,12].

Due to the growing interest in the characterization of endemic plants in pharmaceutical, cosmetic and food industry and as a part of our continuing investigation seeking new ways to enhance the recovery of bioactive substances, different extracts from *Thymus algeriensis* aerial parts were obtained by classical maceration, microwave-assisted extraction and supercritical fluid extraction, and compared by means of their phenolic content by a validated quantitative and analytical high performance liquid chromatography (HPLC-PDA) method. To the best of our knowledge this is the first attempt to characterize this plant species for the presence of these compounds which are well known to exert modulatory effects on biological systems.

To approach this issue, we decided to study the ability of three different extraction techniques on the recovery of the most abundant phenolic secondary metabolites according to an HPLC-PDA method validated in our laboratory [13]. After each extraction, some parameters were optimized in order to improve the phenolic pattern profile in terms of recovery and amount of each constituent. Hydroalcoholic extraction was then performed and compared with supercritical fluid (SFE) and microwave-assisted (MAE) extractions in terms of yield and recovery. In particular, the application of microwaves for heating the solvents and plant tissues in extraction process is known to increase the kinetic of extraction, to reduce extraction time and solvent waste, to promote higher extraction rate and to save costs compared to classical methods [14].

Moreover, SFE furnishes some operational advantages since it works with supercritical solvents with different physicochemical properties such as graduable density, relatively high diffusivity and low viscosity, thereby providing enhanced transport properties and faster extraction rates by means of an easy diffusion through solid materials [15].

To gain an efficient and adequate metabolite recovery, a crucial control and successive optimization of each parameter is necessary. Initially, in our experiments (MAE and SFE) we aimed at limiting thermal degradation phenomena setting the temperature at 40 °C. Then, we modified pressure for SFE, in order to modify the density of the supercritical fluid, and temperature for MAE, to study the impact on phenolic recovery.

Successively, in order to improve the chemical composition knowledge of this plant, the aerial parts were further characterized by color analysis using a device-independent color space (CIELAB parameters) as defined by the "Commission Internationale de l'Eclairage" and specific pigments (carotenoids and chlorophylls) pattern. This comprehensive phytochemical profile could be used to better corroborate the traditional use of this plant.

## 2. Experimental Section

### 2.1. Materials

Chemical standards: gallic acid, catechin, caffeic acid, chlorogenic acid, 4-hydroxy-benzoic acid, vanillic acid, epicatechin, syringic acid, 3-hydroxy-benzoic acid, isovanillin, *p*-coumaric acid, rutin, sinapinic acid, *t*-ferulic acid, naringin, 2,3-dimethoxy-benzoic acid, benzoic acid, *o*-coumaric acid, quercetin, *t*-cinnamic acid, naringenin, carvacrol, harpagoside (all purity >98%) were purchased by Sigma-Aldrich (Milan, Italy). Methanol, chloroform, ethyl acetate and *n*-butanol (HPLC-grade), acetic acid (99%), acetonitrile (HPLC-grade) were obtained from Carlo Erba Reagenti (Milan, Italy). Double-distilled water was obtained using a Millipore Milli-Q Plus water treatment system (Millipore Bedford Corp., Bedford, MA, USA). All extractions were monitored by thin layer chromatography (TLC) performed on 0.2 mm thick silica gel plates (60 F254 Merck) and the spots were detected under an ultraviolet (UV) lamp (at 254 and 365 nm). Column chromatography was carried out using Sigma-Aldrich silica gel (high purity grade, pore size 60 Å, 200–425 mesh particle size).

### 2.2. Plant Material

Samples of full bloom plants were collected from wild population in M’Sila region (Coordinates: 35°42′N 4°33′E), Algeria, in May 2016 and identified by Professor Mohamed Kaabeche (Biology Department, University of Setif 1, Algeria). A voucher specimen has been deposited in the Herbarium of the VARENBIOMOL research unit, University Frères Mentouri Constantine 1. Aerial parts were manually separated, dried at controlled temperature (40 ± 1 °C) in the dark until constant weight. Then plant material was powdered to a uniform granulometry and stored in the dark at –20 °C, in vacuum bags, until extractions and further phytochemical analyses.

## 3. Extraction Procedures

### 3.1. Hydroalcoholic Extraction and Fractionation

The air-dried aerial parts (leaves and flowers, 2.0 kg) of *T. algeriensis* were powdered (slight grinding at controlled temperature, up to 35 °C) and macerated at room temperature with EtOH–H_2_O 70:30, (*v*/*v*) (15 L) for 24 h, four times with fresh solvent. After a filtration step, the extracts were combined, concentrated under reduced pressure, diluted in H_2_O (800 mL) under magnetic stirring and maintained at 4 °C overnight to precipitate chlorophylls. After a second filtration step, the resulting solution was extracted with solvents with increasing polarities (chloroform, ethyl acetate and *n*-butanol). Each extract was dried with anhydrous Na_2_SO_4_, filtered over Chromafil^®^ PET 20/25 (0.2 µm pore size, Machery-Nagel AG, Oensingen, Switzerland) into brown glass vials and concentrated under vacuum (up to 35 °C) to yield the following extracts: CHCl_3_ (7.42 g), EtOAc (4.19 g), *n*-BuOH (33.15 g).

**Figure 1:**
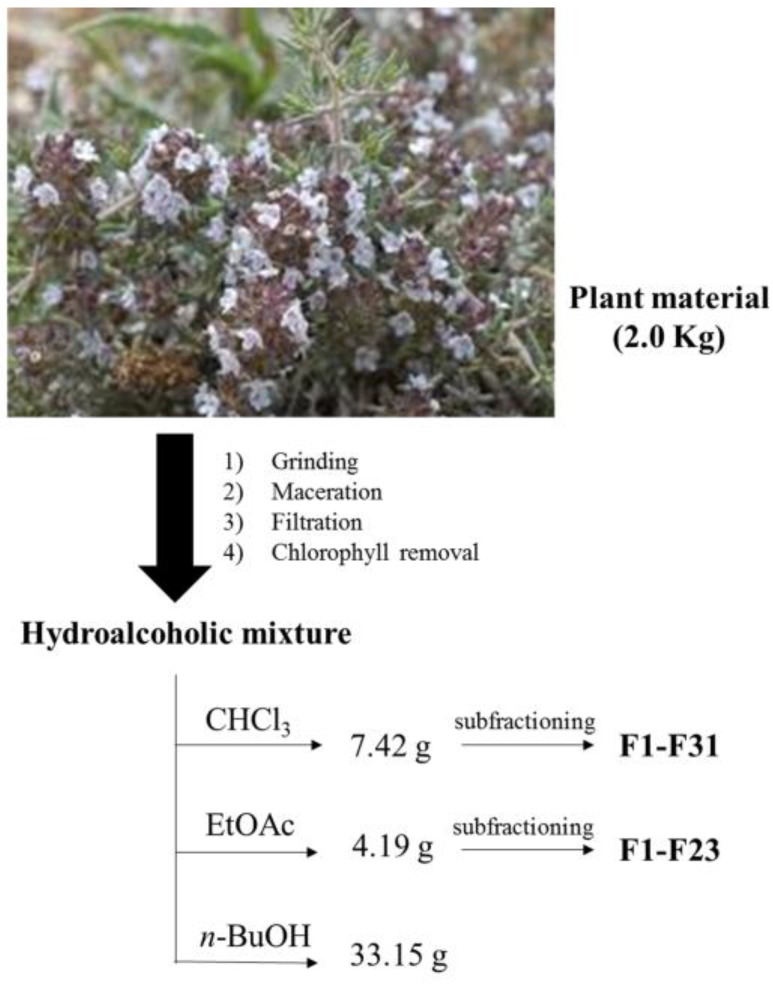
Schematic flowchart performed on *Thymus algeriensis* aerial parts.

The chloroform extract was further fractionated by column chromatography (on silica gel; cyclohexane/diethyl ether, step gradients) to yield 31 fractions (F1–F31), combined according to their TLC profiles. The ethyl acetate extract was also further fractionated by column chromatography (on silica gel; CHCl_3_/MeOH, step gradients) to yield 23 fractions (F1–F23), combined according to their TLC profiles. The extracts/subfractions were collected in a vial at room temperature, the extraction solvent was dried under a gentle N_2_ flow at room temperature and the residue stored at −20 °C until chromatographic analysis (Figure 1).

### 3.2. Supercritical Fluid Extraction (SFE)

The SFE extractor consists of a CO_2_ delivery pump (PU-2080-CO_2_, Jasco, Tokyo, Japan), a thermostatic chamber with a 50 mL extraction column, an UV-Vis detector equipped with high-pressure cell (875-UV, Jasco) and an automatic back pressure regulator (BP-2080 plus, Jasco). A sample of triturated aerial parts (20 g) was packed in a 50 mL SFE extraction bulk. The plant material was exposed to a dynamic extraction at 40 °C for 60 min with a CO_2_ flow-rate of 3 mL/min. Two different pressure values (10 and 30 MPa) were applied in order to modulate supercritical fluid density as also reported in the literature regarding *Thymus*-related species [16]. After filtration over Chromafil^®^ PET 20/25 (0.2 µm pore size, Machery-Nagel AG, Oensingen, Switzerland) into brown glass vials, the extracts (yield 0.73% for 10 MPa and 0.65% for 30 MPa) were collected at room temperature and stored at −20 °C until chromatographic analysis.

### 3.3. Microwave-Assisted Extraction (MAE)

MAE was performed using an automatic Biotage Initiator^TM^ 2.0 (Uppsala, Sweden) characterized by 2.45 GHz high-frequency microwaves and power range 0–300 W. The internal vial temperature was strictly controlled by an infrared (IR) sensor probe. Ground samples were added with water (20:1 *v*:*w*, liquid-to-solid ratio). Then, the suspension was transferred in a 10 mL sealed vessel suitable for an automatic single-mode microwave reactor. MAE was carried out heating by microwave irradiation at 40, 60, 80, 100 or 120 °C for 5, 10 or 15 min and then cooling with pressurized air. After filtration over Chromafil^®^ PET 20/25 (0.2 µm pore size, Machery-Nagel AG, Oensingen, Switzerland) into brown glass vials, the extraction solvent was dried under a gentle N_2_ flow at room temperature. The dried mixtures (yields between 8.3–10.1%) were stored at −20 °C until further chromatographic analysis [17].

### 3.4. HPLC Analysis

HPLC-PDA phenolic pattern was evaluated by the validated method reported in the literature [18], using an HPLC Waters liquid chromatography (model 600 solvent pump, 2996 PDA) and a Phenomenex prodigy ODS(3) 100A 250 mm × 4.6 mm, 5 µm as column. Mobile phase was directly *on-line* degassed by using a Biotech 4CH DEGASI Compact (Onsala, Sweden). Empower v.2 Software (Waters Spa, Milford, MA, USA) was used to collect and analyze data. All extracts were weighted, dissolved in mobile phase and then 20 µL were directly injected into HPLC-PDA system. For over range samples, 1:10 dilution factor was applied. Data are reported as mean ± standard deviation of three independent measurements. The identification of individual compounds was carefully performed on the basis of their retention time (verified also by UV-Vis spectra) by comparison with those of pure standard compounds, without difficulties in peak tracking when multiple substances co-elute (see supplementary Materials, section S1).

## 4. Color Analysis

CIELAB parameters (L*, a*, b*, C*_ab_ and *h*_ab_), as defined by the "Commission Internationale de l'Eclairage", were determined on the powdered aerial parts of *T. algeriensis* using a colorimeter X-Rite SP-62 (X-Rite Europe GmbH, Regensdorf, Switzerland), equipped with a D65 illuminant and an observer angle of 10°. Color description was based on three parameters: L* that defines the lightness and varies between 0 (absolute black) and 100 (absolute white), a* that measures the greenness (−a*) or the redness (+a*) and b* that measures the blueness (−b*) and the yellowness (+b*). C*_ab_ (chroma, saturation) expresses a measure of color intensity and *h*_ab_ (hue, color angle) is the attribute of appearance by which a color is identified according to its resemblance to red, yellow, green, or blue, or a combination of two of these attributes in sequence. Cylindrical coordinates C*_ab_ and *h*_ab_ are calculated from the parameters a* and b* using the equations C*_ab_ = (a*^2^ + b*^2^)^½^ and *h*_ab_ = tan^−1^(b*/a*) [19]. Three different powdered *T. algeriensis* aerial parts samples were analyzed. The results are expressed as the mean value ± standard deviation (SD).

## 5. Carotenoids and Chlorophylls Analysis

The total carotenoids and chlorophylls a and b analysis in *Thymus* sample was performed according to Solovchenko with some modifications [20]. The sample was homogenized with mortar and pestle in 5 mL of chloroform-methanol (2:1, *v*/*v*) containing 0.01% butylated hydroxytoluene (BHT) to inhibit peroxidation process. Moreover, homogenization was carried out with 50 mg of MgO to prevent chlorophyll pheophytinization. The homogenate was passed through a paper filter and after, distilled water was added to the amount of 0.2 of the extract volume. Finally, the mixture was centrifuged in glass tube test for 18 min at 3000 g at 10 °C to complete separation of chloroform fraction from methanol/aqueous one. The chloroform phase (lower phase) contained the hydrophobic molecules (chlorophylls, carotenoids, lipids, etc.), while the methanol-water phase (upper phase) contained the hydrophilic molecules. Absorption spectrum of the chloroform phase was recorded with a Beckman Coulter DU 800 instrument in the range of 350–800 nm with a spectral resolution of 0.5 nm at a temperature of 20 °C. Both chlorophylls and the total carotenoid contents were determined using absorption coefficient according to Wellburn (1994) [21]. Equations to determine the concentrations of chlorophyll a (C_a_) and b (C_b_), as well as total carotenoid (C_tot_) contents are reported below:

C_a_= 11.47 A_665.6_ − 2A_647.6_;

C_b_= 21.85 A_647.6_ – 4.53 A_665.6_;

C_tot_= (1000 A_480_ – 1.33C_a_ – 23.93 C_b_)/202.

The data are reported as means of three replications and expressed as µg/mg DW (dry weight) ± SD (standard deviation).

## 6. Results and Discussion

### 6.1. Hydroalcoholic Extracts and Subfractions

The aim of this analysis was to carry out a qualitative and quantitative study of the different extracts or subfractions of *T. algeriensis* aerial parts, and also to compare the extractive procedures in order to develop extraction methods with better yields (Table 1). The use of different solvents with increasing polarity led to a preliminary but metabolite-oriented purification. Successive subfractioning of CHCl_3_ and EtOAc extracts highlighted better the presence of specific secondary metabolites based on the results obtained by means of our validated high performance liquid chromatography-photodiode array detector (HPLC-PDA) procedure.

Ethyl acetate extract was the richest of phenolic constituents reaching *p*-coumaric acid and benzoic acid the highest concentration in some isolated fractions (40.62 µg/g and 5.71 µg/g, respectively), and catechin having the highest concentration in a successive isolated one (6.23 µg/g).

Finally, eleven compounds were identified in chloroform extract, among which naringenin and benzoic acid resulted with the highest concentrations in the fractions F16 and F24 (8.97 µg/g and 10.92 µg/g, respectively), and only epicatechin was identified in the fraction F30 with the concentration of 6.78 µg/g.

Nine compounds were identified in the *n*-butanol fraction, including three flavonoids among which epicatechin had the highest concentration (48.03 µg/g), while of the six phenolic acids present in this fraction, *o*-coumaric acid resulted in the highest concentration (9.83 µg/g).

### 6.2. Supercritical Fluid Extraction (SFE)

The aim of this experiment was to study the influence of the operating pressure parameter on the kinetics of the supercritical extraction process, in order to optimize the operative conditions and to compare the performance with the other extraction methods, using the secondary metabolites profile as discriminant marker.

We carried out two separate plant sample extractions at a fixed temperature of 40 °C, operating with different pressure values (10 MPa and 30 MPa) in order to find the leading coordinates towards the best extraction yield. We found that the increasing pressure did not improve either yield or the phenolic recovery, with only slight effects on the vanillic acid amount. According to the quality and the quantity of metabolites, the more interesting results were obtained under the following operating conditions: 40 °C for the temperature and 30 MPa for the pressure (Table 2). Collectively, this method afforded low amounts for phenolic compounds from *T. algeriensis* aerial parts.

### 6.3. Microwave-Assisted Extraction (MAE)

Design and optimization of the microwave-assisted extraction of this plant were performed keeping in mind the impact of different parameters (temperature, time, and solvent volume) on the recovery of main metabolites profile used as discriminant marker.

The choice of the solvent, to afford the best extraction yield, is one of the most important steps for the development of an extraction method. In order to obtain good results, a preliminary microwave-assisted extraction based on water as a polar solvent (solvent volume: 20 mL; plant material: 1 g; extraction time: 10 min) was applied and the best extraction temperature ranging from 40 to 120 °C was evaluated by means of the results provided by HPLC method (see supplementary Materials, section S2). Once selected the best temperature condition (100 °C), to obtain the most diversified extract in phenolics, we tried extraction times of 5 and 15 min, obtaining the best extraction yield with an extraction time of 15 min in the same solvent (water). Finally, we have applied these conditions to select the best extraction solvent. The aqueous extract resulted richer in plant secondary phenolic metabolites than the EtOH/H_2_O (70/30, v/v) extract, obtained in the same conditions (Table 3).

The richest phenolic pattern was obtained in the previously selected conditions (temperature: 100 °C, time: 15 min) using water as the solvent. Several compounds were identified in this extract, whose composition is reported in Table 3, which contained a complex mixture of plant secondary metabolites belonging to the chemical classes of phenolic acids and flavonoids, both known for their pharmacological activities.

The phenolic acids were identified as gallic acid, chlorogenic acid, vanillic acid, syringic acid, 3-hydroxybenzoic acid, *p*-coumaric acid, sinapinic acid, *t*-ferulic acid, benzoic acid, *o*-coumaric acid. Benzoic acid displayed the highest concentration (4145.75 µg/g) followed by epicatechin (246.752 µg/g), chlorogenic acid (1745.98 µg/g), syringic acid (615.20 µg/g), naringin (376.60 µg/g), catechin (359.80 µg/g), and *o*-coumaric acid (341.55 µg/g). Flavonoids and phenolic compounds are the most important groups of secondary metabolites and bioactive compounds in plants [22,23]. Two important trends could be also extrapolated from the impact of the increasing temperature on the recovery: first, some compounds (chlorogenic acid, *t*-ferulic acid, quercetin, isovanillin, epicatechin, syringic acid, catechin and *p*-coumaric acid) reached the highest amount until 100 °C and then their concentrations tended to diminish at higher temperatures. Secondly, other compounds (vanillic acid, gallic acid, sinapinic acid, naringin, 2,3-dimethoxybenzoic acid, and *o*-coumaric acid) had their maximum recovery at lower temperature and extraction time.

The experimental results show that aqueous extraction, assisted by microwave at temperature 100 °C and for a time of 15 min, produced a phenolic-enriched extract of *Thymus algeriensis* aerial parts.

### 6.4. Color Analysis

Dry samples of *Thymus algeriensis* aerial parts were blended in a mixer and further homogenized in a mortar. The samples of blended powdered dry leaves of *Thymus* showed a nonhomogeneous color in which three different shades could be evidenced, a pale beige, a pale pink and a pale green. Conversely, the mortar homogenized samples displayed a more homogeneous light brown color characterized by two only pale nuances, a pale green and an undefined pale shade among beige, pink and orange. The tristimulus colorimetry was employed in this study to evaluate the color properties: the colorimetric data and the mean value, completed by the standard deviation, of three different samples, are reported in Table 4.

**Figure 2:**
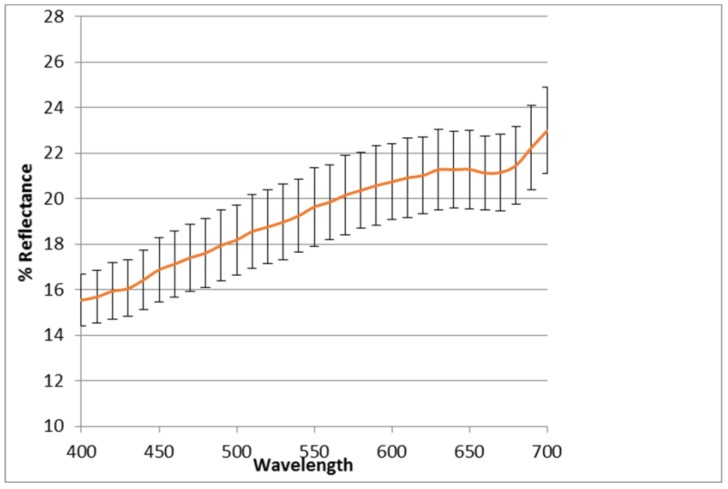
Reflectance analysis of *Thymus algeriensis* aerial parts.

It is not possible to give an interpretation for comparison of this CIELAB colorimetric analyses, because only few data are available in literature about colorimetric studies on aerial parts in general, and no data are disposable for this plant in particular. Moreover, as known, the colorimetric parameters are deeply influenced by several different factors [24,25], among which storage temperature, humidity, light exposition, besides the standard conditions applied, according to the Commission Internationale de l'Éclairage (CIE). In this work a D65 illuminant, sunlight simulating, with an observer angle of 10°, was used. Reflectance curve is reported in Figure 2. Only a weak yellow parameter (light positive b*) can be shown, in line with the phenolic profile denoted by the HPLC analysis.

### 6.5. Pigments Determination (Total Carotenoids, Chlorophyll a and Chlorophyll b)

**Figure 3:**
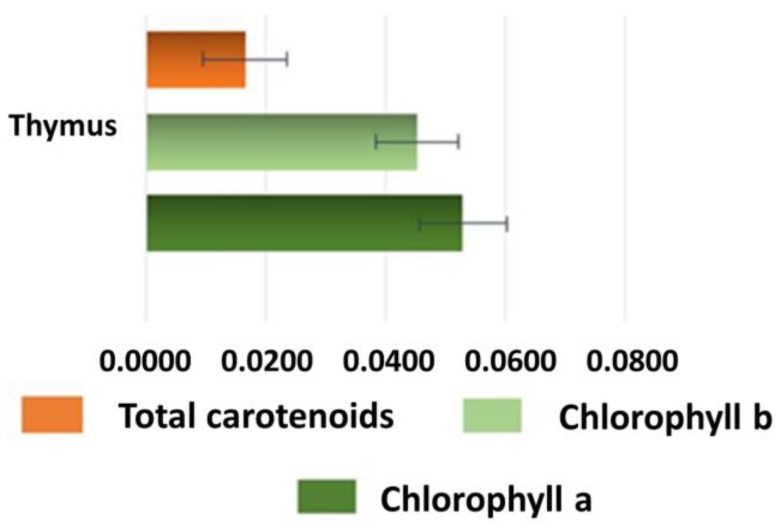
Pigments concentration in *T. algeriensis* aerial parts (µg/mg ± SD).

*T. algeriensis* aerial parts plant displayed significant differences in analyzed pigments concentration. It is possible to notice that chlorophyll a content (0.0529 ± 0.0072 µg/mg) is higher compared with the other pigments (chlorophyll b =0.0452 ± 0.0069 µg/mg and carotenoids 0.0165 ± 0.007 µg/mg) (*p* < 0.002) (Figure 3), but chlorophyll b was present in good proportion, as expected in shade plants. Generally, the concentration of chlorophyll a (Chl a) is two to three times the concentration of chlorophyll b (Chl b) due to the importance of chlorophyll a as the primary pigment in photosynthesis. However, a greater proportion of Chl b improved light-collection ability of the leaf in the region of far-red light. In this plant, the value of the Chl a/b ratio was 1.17, indicating a high chlorophyll a content. Chl a/b ratio can be a useful indicator of nitrogen partitioning within a leaf and this ratio is predicted to respond to light and nitrogen availability. In particular, Chl a/b ratio should increase with increasing irradiance at a given nitrogen availability [26]. Regarding total carotenoid concentration, our finding showed an amount about three times lower than chlorophyll a (0.0165 ± 0.007 µg/mg and 0.0529 ± 0.0072 µg/mg, respectively) (Figure 3). High carotenoid contents are revealed under high insolation level, because in this case they act as protectors from photoinhibition [26]. The quali-quantitative evaluation of these ubiquitous bulk substances is of crucial interest not only due to their role in the protection against oxidative stress [27], but also for their interference with biological assays generating false positive and negative results [28].

## 7. Conclusions

The study of endemic plants could support their ethnobotanical use by means of a wide comprehension of the qualitative and quantitative phytochemical profile. After the evaluation of color parameters and pigment content of the aerial parts of *T. algeriensis*, we developed an HPLC method with diode array detection for the quantitative and qualitative estimation of phenolic compounds obtained by different extraction methods. Three extraction methods have been carried out in this work for the purpose of developing extractions under the best conditions. The results of the present study revealed important data regarding the phenolic composition of *Thymus algeriensis* aerial parts up to now; several phenolic compounds known for their pharmacological properties were identified and quantified in different extracts of this plant. MAE was shown to be the best-performing procedure. The presence of a significant amount of respective bioactive components in this plant and the variation of quantity based on the polarity of the solvent used for the extraction process ensured its unequivocal recommendation for use in the pharmaceutical and nutraceutical sector.

## Figures and Tables

**Table 1:** Phenolic profile of main subfractions of *Thymus algeriensis* aerial part extracts obtained by maceration.

Identified Compound	Content (µg/g ± SD, DW)						
	F16 (CHCl_3_)	F24 (CHCl_3_)	F30 (CHCl_3_)	F13 (EtOAc)	F22 (EtOAc)	F27 (EtOAc)	*n*-BuOH
**Catechin**	1.12 ± 0.01					6.23 ± 0.05	
**4-Hydroxy-benzoic acid**					16.31 ± 0.91	3.61 ± 0.30	0.66 ± 0.02
**Vanillic acid**	5.17 ± 0.11	0.23 ± 0.01			0.22 ± 0.01		
**Epicatechin**			6.78 ± 0.12	0.55 ± 0.01			48.03 ± 2.98
**Syringic acid**							1.93 ± 0.11
***p*-Coumaric acid**				1.26 ± 0.81	40.62 ± 3.01	1.63 ± 0.88	1.70 ± 0.58
**Rutin**	0.57 ± 0.02						4.52 ± 0.41
***t*-Ferulic acid**	0.20 ± 0.01	0.23 ± 0.01			1.46 ± 0.13	0.84 ± 0.01	0.55 ± 0.01
**Naringin**		0.16 ± 0.01		4.02 ± 0.39	0.46 ± 0.01		
**2,3-Dimethoxy-benzoic acid**	6.51 ± 0.59				7.51 ± 0.47		3.52 ± 0.20
**Benzoic acid**		10.92 ± 1.21		5.71 ± 0.47			
***o*-Coumaric acid**						1.03 ± 0.09	9.83 ± 0.87
**Naringenin**	8.97 ± 0.74	0.90 ± 0.03					0.47 ± 0.01
**Carvacrol**	0.43 ± 0.01						
**Total**	22.97 ± 1.01	12.44 ± 1.03	6.78 ± 0.12	11.54 ± 0.99	66.58 ± 2.70	13.34 ± 1.12	71.21 ± 2.40

DW: dry weight.

**Table 2:** Phenolic profile of *T. algeriensis* aerial part extracts obtained by supercritical fluid extraction obtained at two discrete pressure values.

Identified Compound	Content (µg/g ± SD, DW)	
	40 °C, 10 MPa	40 °C, 30 MPa
**Gallic acid**	0.10 ± 0.01	0.10 ± 0.01
**Catechin**	0.05 ± 0.01	0.05 ± 0.01
**Vanillic acid**		0.18 ± 0.02
**Epicatechin**	0.15 ± 0.02	0.15 ± 0.01
**Isovanillin**	1.49 ± 0.09	1.48 ± 0.06
***p*-Coumaric acid**	0.17 ± 0.02	0.14 ± 0.01
**Naringin**	0.06 ± 0.01	0.06 ± 0.01
**Harpagoside**	0.10 ± 0.01	0.10 ± 0.01
**Total**	2.12 ± 0.10	2.26 ± 0.10

DW: dry weight.

**Table 3:** High performance liquid chromatography-photodiode array detector (HPLC-PDA) analysis of the phenolic profile of *Thymus algeriensis* microwave-assisted extracts.

Identified Compound	Content (µg/g ± SD, DW)							
	40 °C, 10 min, water	60 °C, 10 min, water	80 °C, 10 min, water	100 °C, 10 min, water	120 °C, 10 min, water	100 °C, 5 min, water	100 °C, 15 min, water	100 °C, 15 min, EtOH/H_2_O 50:50
**Gallic acid**	36.51 ± 0.23	72.84 ± 0.36	24.57 ± 0.21	28.05 ± 0.19	20.31 ± 0.20	9.70 ± 0.09	37.97 ± 0.25	
**Catechin**				46.69 ± 0.33	238.91 ± 1.66	45.23 ± 0.18	359.80 ± 1.98	
**Chlorogenic acid**	118.34 ± 1.01	1203.98 ± 5.29	1090.00 ± 4.77	1766.64 ± 5.13	930.04 ± 3.99	1570.40 ± 4.33	1745.98 ± 5.65	
**4-Hydroxy-benzoic acid**			18.38 ± 0.20					37.38 ± 0.66
**Vanillic acid**		37.56 ± 0.71	50.17 ± 0.98	9.73 ± 0.12		176.74 ± 1.02	23.92 ± 0.66	
**Epicatechin**			813.21 ± 2.29	1675.51 ± 3.95	821.40 ± 3.02	200.62 ± 1.15	2462.75 ± 2.00	66.11 ± 0.99
**Syringic acid**		17.86 ± 0.99	52.22 ± 1.39	315.18 ± 3.00		747.63 ± 3.23	615.20 ± 4.03	
**3-Hydroxy-benzoic acid**		243.31 ± 2.21	57.94 ± 0.98	212.38 ± 1.75	70.62 ± 0.67	75.06 ± 0.60	166.73 ± 1.02	19.82 ± 0.51
**Isovanillin**		223.75 ± 1.97	248.12 ± 1.88	271.11 ± 2.04	59.05 ± 0.77	68.21 ± 0.67	40.42 ± 0.78	200.37 ± 1.09
***p*-Coumaric acid**	15.78 ± 0.13	14.07 ± 0.29	21.65 ± 0.28	40.00 ± 0.32	23.05 ± 0.27	18.82 ± 0.23	106.99 ± 0.77	62.02 ± 0.19
**Rutin**	39.70 ± 0.23	375.42 ± 1.01	37.63 ± 0.65	89.45 ± 0.69		16.22 ± 0.09	196.89 ± 1.00	55.32 ± 0.55
**Sinapinic acid**	4.96 ± 0.13	31.58 ± 0.60	5.37 ± 0.17	5.53 ± 0.17	5.26 ± 0.18	29.64 ± 0.60	46.20 ± 0.63	8.48 ± 0.10
***t*-Ferulic acid**		19.89 ± 0.99	14.22 ± 0.50	27.56 ± 0.52	21.72 ± 0.54	1869.72 ± 2.01	140.64 ± 0.73	
**Naringin**	89.63 ± 0.98	385.92 ± 2.12	179.46 ± 2.03	29.64 ± 0.23	31.31 ± 0.24	132.37 ± 1.00	376.60 ± 2.77	1611.91 ± 2.49
**2,3-dimethoxy-benzoic acid**	520.37 ± 2.02	1212.75 ± 5.84	222.84 ± 1.95					507.77 ± 2.39
**Benzoic acid**	1455.82 ± 3.39	4896.51 ± 5.77	2425.66 ± 4.23	2393.82 ± 4.78	2697.97 ± 5.00	3889.74 ± 4.77	4157.75 ± 4.67	
***o*-Coumaric acid**	227.74 ± 1.04	348.86 ± 2.20	37.66 ± 0.45	23.51 ± 0.29	30.27 ± 0.28	812.41 ± 2.00	341.55 ± 1.17	163.21 ± 0.99
**Quercetin**			63.04 ± 0.20	74.71 ± 0.23	59.52 ± 0.20	68.60 ± 0.28	180.72 ± 0.77	
**Total**	2508.85 ± 5.36	9084.30 ± 8.12	5362.13 ± 4.99	7009.51 ± 8.54	5009.41 ± 4.12	9731.14 ± 7.98	11000.12 ± 9.96	2732.38 ± 5.88

DW: dry weight.

**Table 4:** Color analysis using a device-independent color space (CIELAB parameters), as defined by the "Commission Internationale de l'Eclairage", data of *Thymus algeriensis* aerial parts.

CIELAB Parameters	Mean Value	SD
L*	51.25	1.91
a*	1.09	0.36
b*	5.53	0.69
C*_ab_	5.64	0.72
*h*_ab_	78.90	3.28

## Supplementary Materials

The following are available online, Section S1: Chemical standards resolution in HPLC-PDA method and gradient elution profile, Section S2: HPLC-PDA chromatograms obtained for MAE optimization.
