# Supplementary material for: Qualitative and Quantitative Phytochemical Analysis of Different Extracts from Thymus algeriensis Aerial Parts
Source: Molecules. 2018 Feb 20;23(2):463. doi: 10.3390/molecules23020463 (PMC6017170; doi:10.3390/molecules23020463)

Supplementary materials

Qualitative and quantitative phytochemical analysis of different extracts from *Thymus algeriensis* aerial parts

Nassima Boutaoui[a], Lahcene Zaiter[a], Fadila Benayache[a], Samir Benayache[a], Simone Carradori[b,*], Stefania Cesa[c], Anna Maria Giusti[d], Cristina Campestre[b], Luigi Menghini[b], Denise Innosa[e], Marcello Locatelli[b]

[a]*Unité de recherche Valorisation des Ressources Naturelles, Molécules Bioactives et Analyses Physicochimiques et Biologiques. Université Frères Mentouri, Constantine 1, Route d'Aïn El Bey, 25000, Constantine, Algérie.*

[b]*Department of Pharmacy, University "G. d'Annunzio" of Chieti-Pescara, Via dei Vestini 31, 66100 Chieti, Italy.*

[c]*Dipartimento di Chimica e Tecnologie del Farmaco, Sapienza Università di Roma, P.le Aldo Moro 5, 00185 Rome, Italy.*

[d]*Dipartimento di Medicina Sperimentale, Sapienza Università di Roma, P.le Aldo Moro 5, 00185 Rome, Italy.*

[e]*Facoltà di Bioscienze e tecnologie agro-alimentari e ambientali, Università di Teramo, Via Renato Balzarini 1, 64100 Teramo, Italy.*

Section S1. Chemical standards resolution in HPLC-PDA method and gradient elution profile

HPLC analysis

HPLC-PDA analyses were performed by a validated method reported in literature [Locatelli et al. 2017] using an HPLC Waters liquid chromatography (model 600 solvent pump, 2996 PDA). Mobile phase was directly degassed *on-line* using a Biotech 4CH DEGASI Compact (Onsala, Sweden). Empower v.2 Software (Waters Spa, Milford, MA, USA) was used to collect and analyze data. The analyses were carried out using gradient elution mode on a C18 reversed-phase column (Prodigy ODS(3), 4.6 x 150 mm, 5 µm; Phenomenex, Torrance, CA), thermostated at 30 °C (± 1 °C). The gradient elution was achieved by a solution of water-acetonitrile (93:7 ratio, with 3% of acetic acid) as initial conditions, and the complete separation was achieved in 60 min.

Chemical standards chromatogram (@ 278 nm as example of wavelength in which all compounds show absorbance) for the analytes, with a table reporting the retention times and the maximum wavelengths used for the quantitative analyses. A table with gradient elution program used for the analyses was also reported.

Gradient elution:

TIME (min)	FLOW (mL min^{-1})	%A	%B
0	1	93	7
0.1		93	7
30		72	28
38		75	25
45		2	98
47		2	98
48		93	7
58		93	7

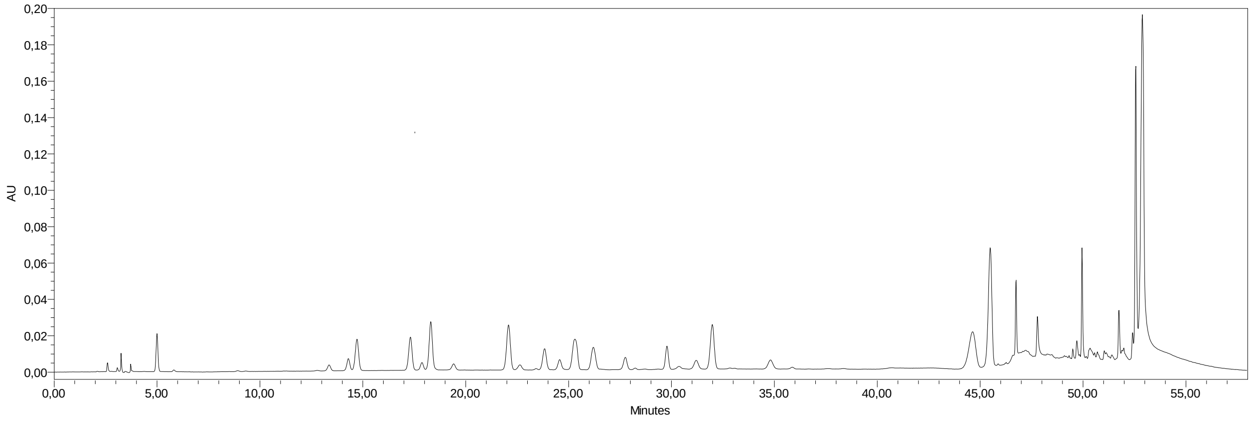

Limit of Detection (LOD) and Limit of Quantification (LOQ)

The limit of detection (LOD, S/N = 3) was 0.075 µg/mL for each analyte. The LOQ (S/N = 10) was 0.25 µg/mL for each analyte at the corresponding maximum wavelength.

Precision and Trueness intra and inter-day

The intra-day precision (RSD%,) of the HPLC determination was in the range 0.5 ÷ 12.3% for the studied phenolics at three quality control (QC) levels within the linearity range; while the trueness was in the range 11.5 ÷ -12.5% at the same QC concentration level. The inter-day precision (RSD% intermediate precision) was in the range 1.3 ÷ 11.8% while the trueness was in the range 12.9 ÷ -12.0%.

Parallelism Test

In order to evaluate the effect of dilution onto the correct quantitative analyses, parallelism test was made. In this test, standard solution with analytes concentration strictly major to the highest calibration point was subjected to a dilution with mobile phase. After quantification on the calibration curve, Bias% and precision values were calculated and the result attests that the samples dilution with mobile phase doesn’t affect the final dosage.

Analytes, retention times, and maximum wavelengths used for quantitative analyses:

**Analytes**	**Retention times (min)**	**λ max**
Gallic acid	4.99	271 nm
Catechin	13.36	278 nm
Chlorogenic acid	14.29	324 nm
4-hydroxybenzoic acid	14.71	256 nm
Vanillic acid	17.31	260 nm
Epicatechin	18.30	278 nm
Syringic acid	18.50	274 nm
3-hydroxybenzoic acid	19.41	275 nm
isovanillin	22.08	278 nm
p-coumaric acid	22.65	310 nm
Rutin	25.38	256 nm
Sinapinic acid	26.18	324 nm
t-ferulic acid	27.75	315 nm
Naringin	29.78	285 nm
2,3-dimethoxybenzoic acid	30.36	299 nm
Benzoic acid	31.20	275 nm
o-coumaric acid	34.81	276 nm
Quercetin	40.57	367 nm
Harpagoside	45.49	280 nm
t-cinnamic acid	45.87	276 nm
Naringenin	46.74	290 nm
Carvacrol	49.95	275 nm

Section S1. HPLC-PDA chromatograms obtained for MAE optimization

40 °C, WATER, 10 min

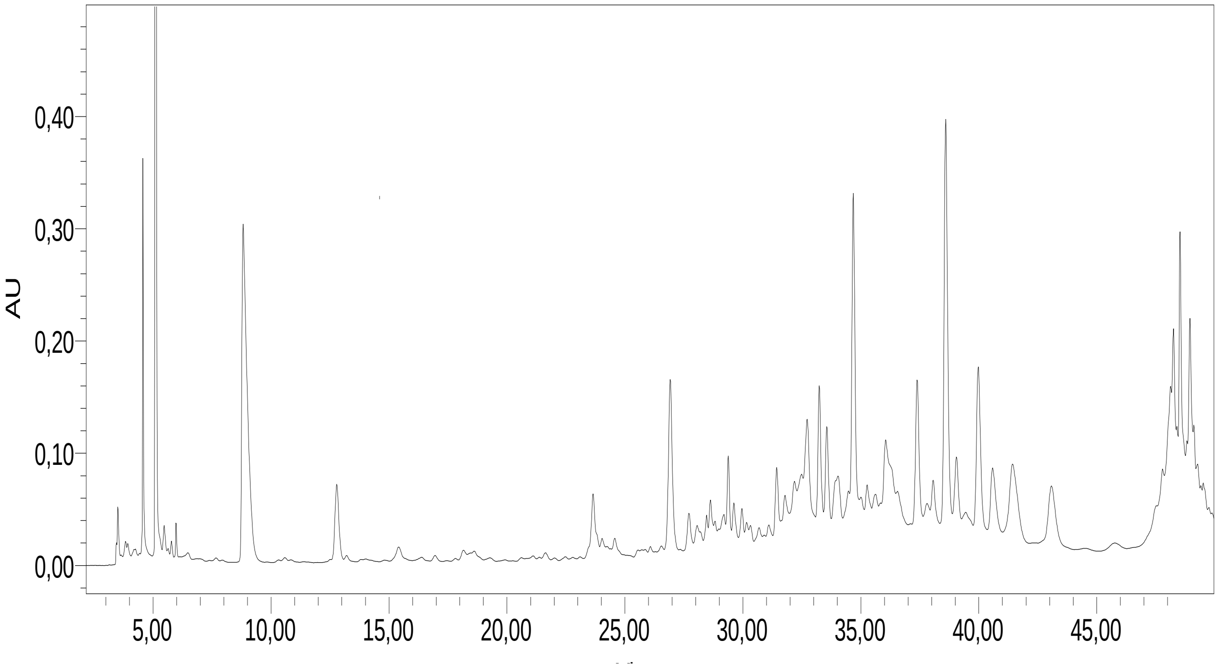

60 °C, WATER, 10 min

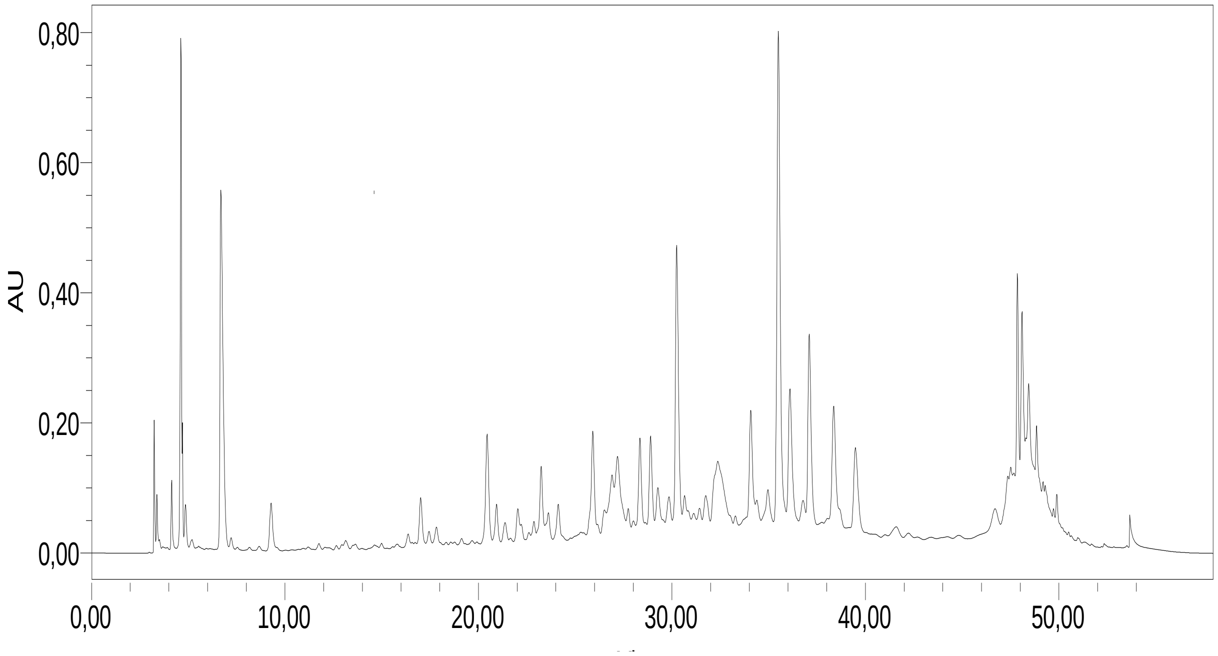

80 °C, WATER, 10 min

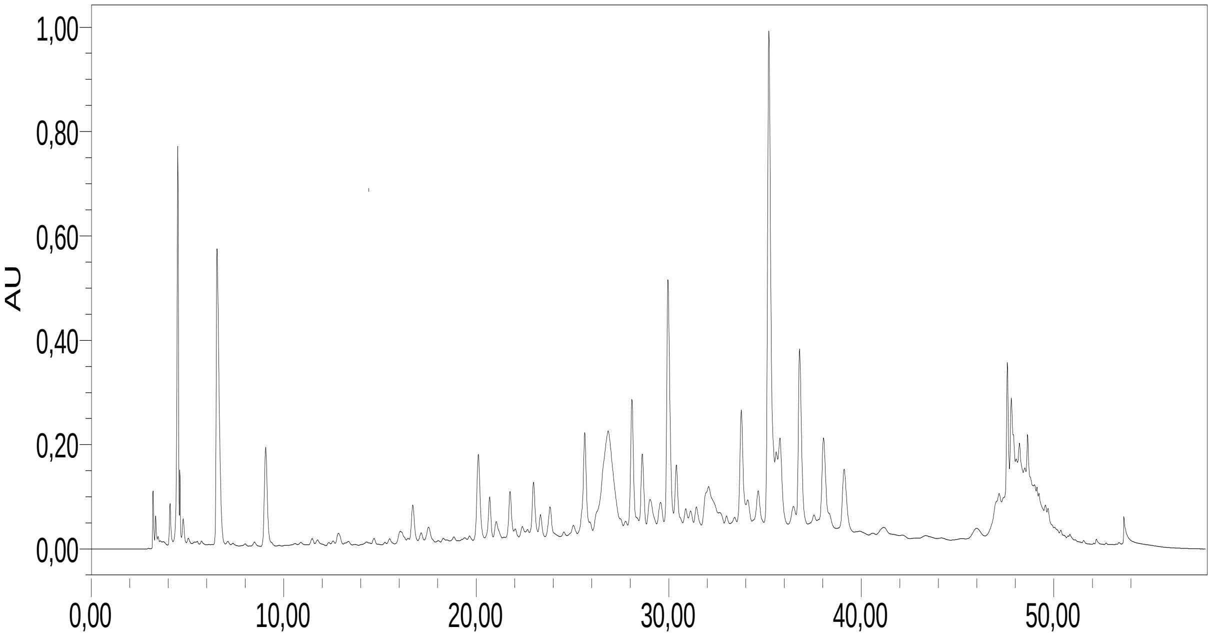

100 °C, WATER, 10 min

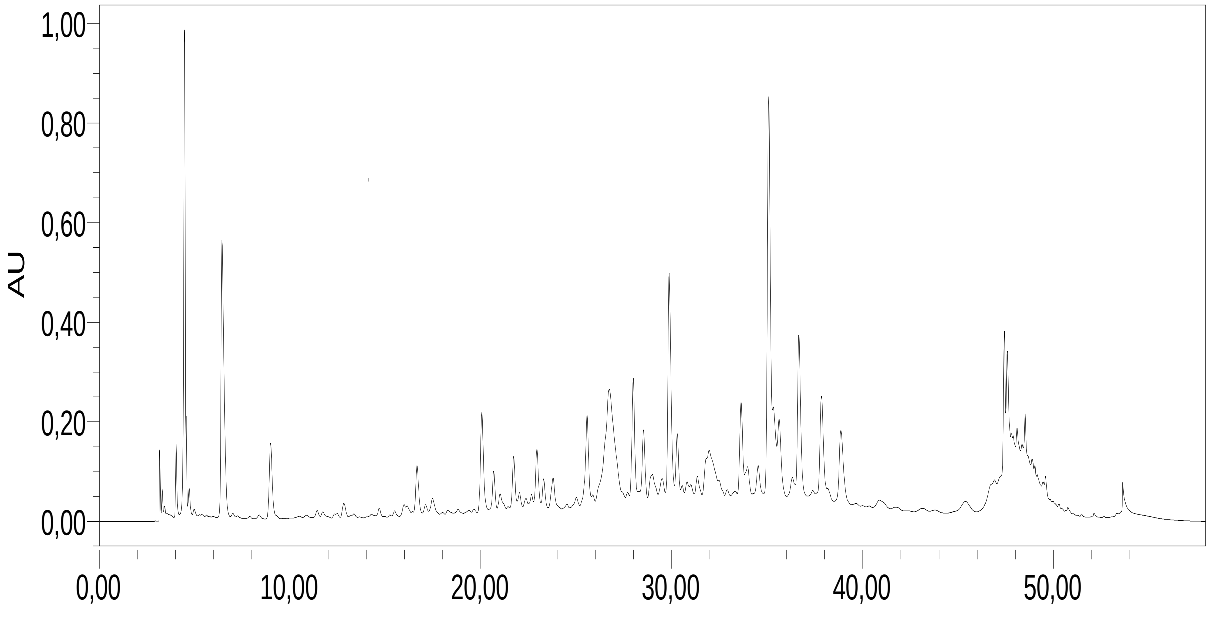

120 °C, WATER, 10 min

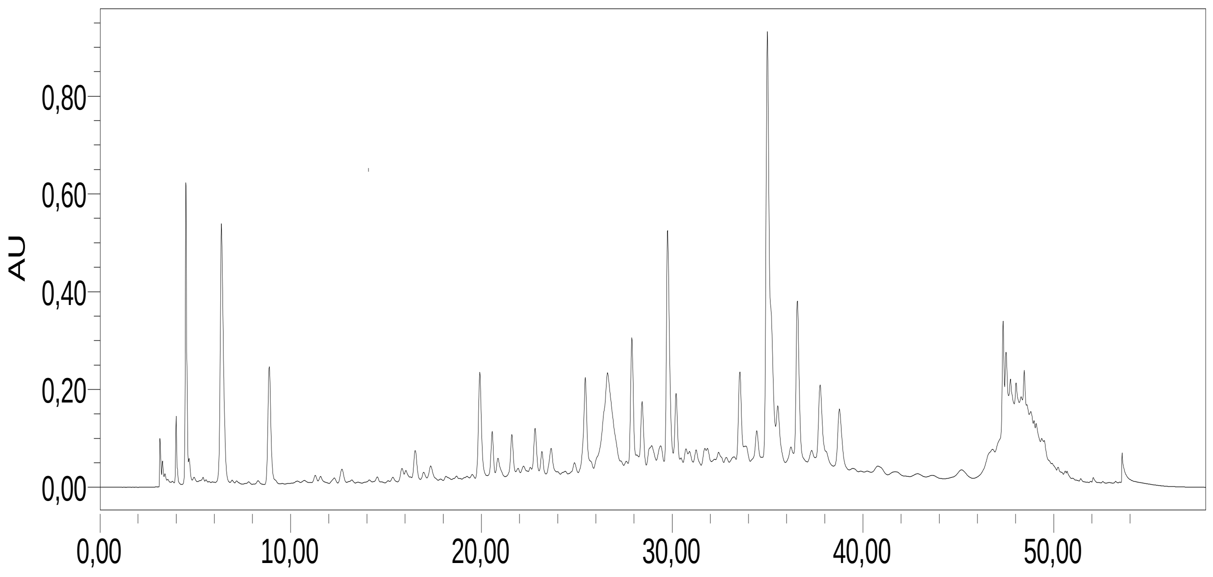

100 °C, WATER, 5 min

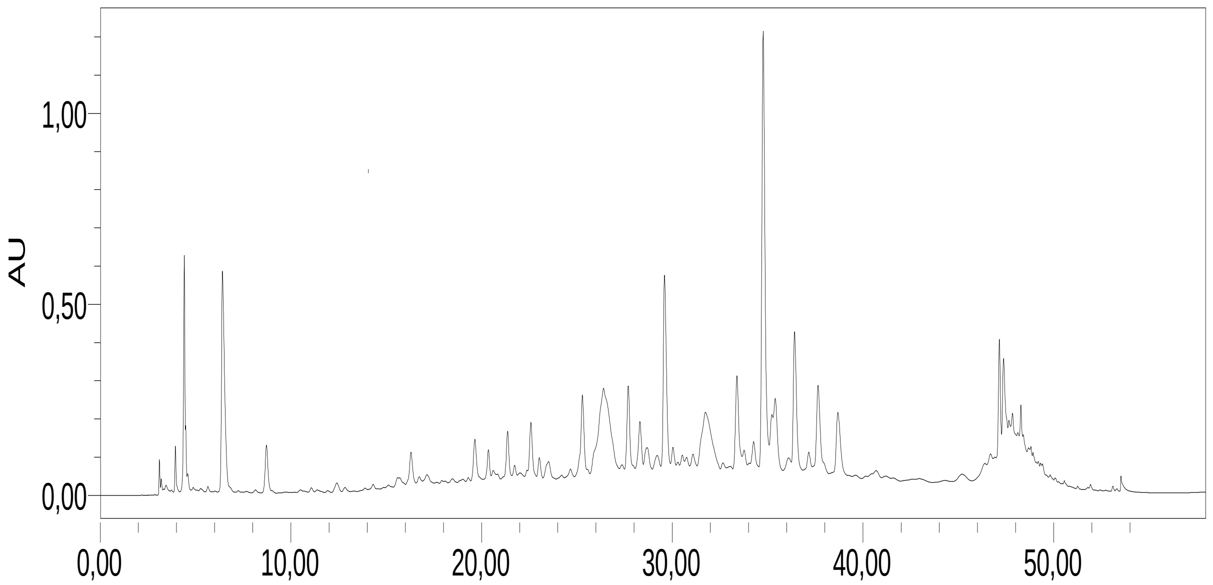

100 °C, WATER, 15 min

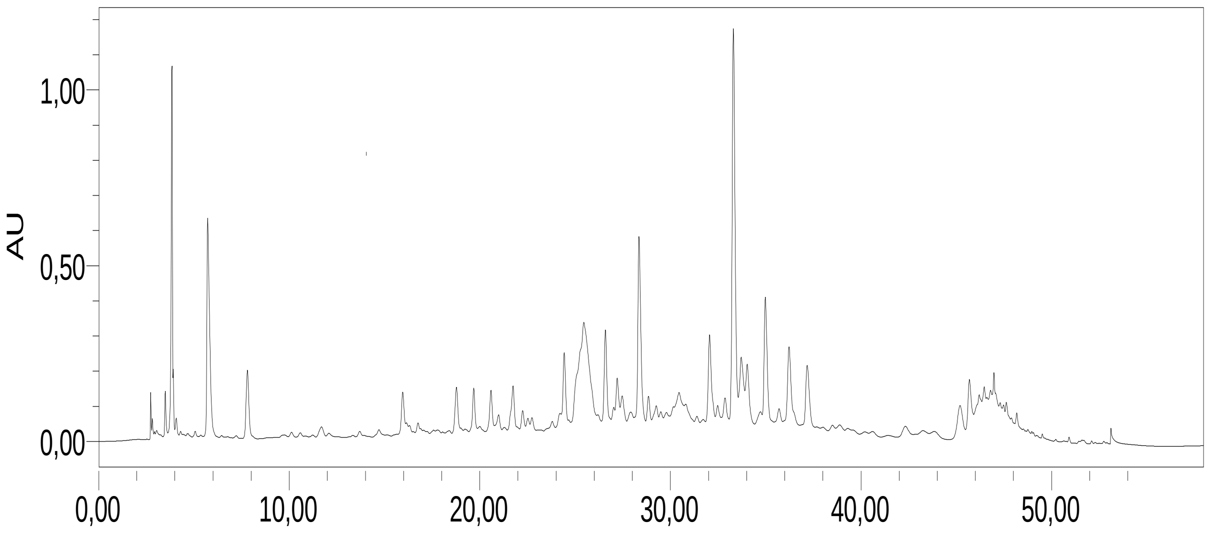

100 °C, WATER/ETHANOL, 15 min

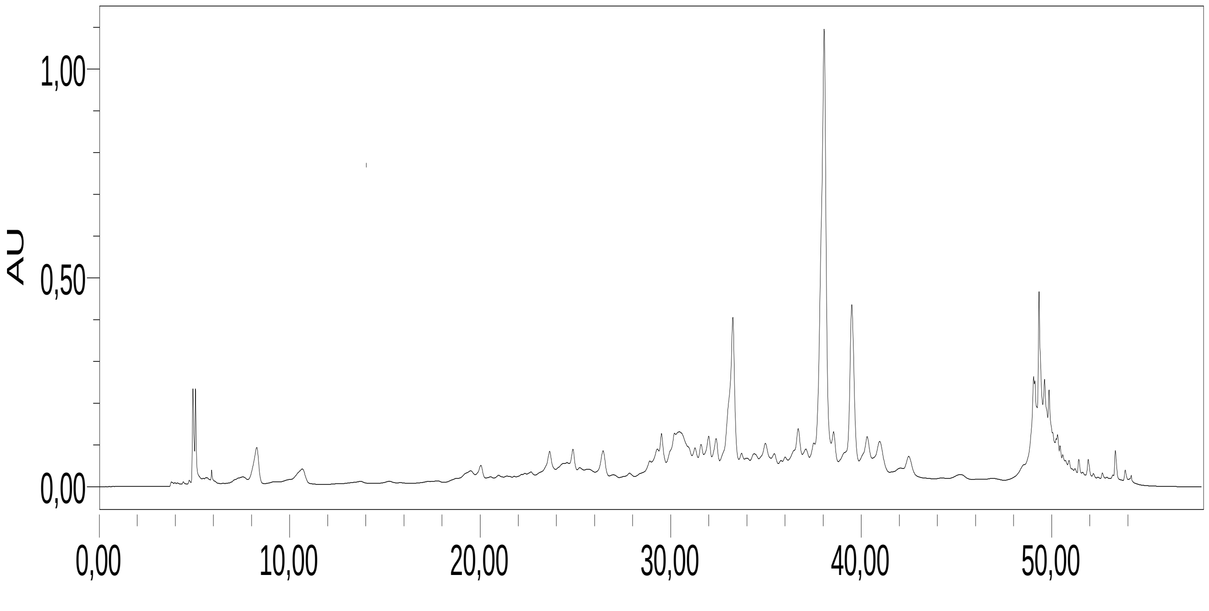

Supplement: Supplementary file 1 [file molecules-23-00463-s001.pdf]
